# Fatal Complications During Photodynamic Bone Stabilization: A Case Report

**DOI:** 10.7759/cureus.88686

**Published:** 2025-07-24

**Authors:** George Mina, Taizoon Q Dhoon, Ramin Rahimian, Shermeen Vakharia, Govind Rajan

**Affiliations:** 1 Anesthesiology and Perioperative Medicine, University of California Los Angeles, Los Angeles, USA; 2 Anaesthesiology, University of California, Irvine, Health, Orange, USA; 3 Anesthesiology and Perioperative Medicine, University of California, Irvine, Health, Orange, USA; 4 Anesthesiology and Perioperative Medicine, University of California, Irvine, Medical Center, Orange, USA

**Keywords:** balloon insufflation, cardiac arrest, cardiopulmonary collapse, debris embolism, extra-corporeal membrane oxygenation, fat embolism, photodynamic bone stabilization, photodynamic implant, pulmonary embolism, venous-arterial extra-corporeal membrane oxygenation

## Abstract

Photodynamic bone stabilization (PBSS) is a minimally invasive technique used in patients with metastatic bone disease who are poor traditional surgery candidates. Although the procedure has a high success rate, we present a fatal case of cardiopulmonary collapse following balloon insufflation during PBSS. The patient had advanced malignancy and preexisting cardiopulmonary conditions and ultimately experienced pulseless electrical activity and hypoxemia. We propose that the likely etiology is embolic debris from medullary reaming. This case highlights the need for perioperative risk stratification, vigilant intraoperative monitoring, and heightened awareness of fatal embolic complications in high-risk patients undergoing PBSS.

## Introduction

Minimally invasive photodynamic bone stabilization (PBSS) is an emerging alternative to traditional orthopedic fixation techniques, particularly for patients with pathological fractures due to metastatic bone disease [1,2]. This system utilizes a balloon catheter filled with a photopolymerizable monomer, which is subsequently hardened in situ, offering both mechanical support and reduced surgical trauma. Compared to intramedullary nailing or cemented arthroplasty, PBSS is a less invasive technique and is increasingly used in older patients, especially with advanced malignancy [3].

However, while PBSS is generally considered safe, isolated reports have emerged describing serious intraoperative complications, including severe hypotension, hypoxia, and cardiovascular collapse [3,4]. These events may result from marrow or fat embolism during reaming or balloon insufflation, mechanisms reminiscent of fat embolism syndrome (FES) and bone cement implantation syndrome (BCIS), which are well-recognized complications of procedures such as intramedullary nailing and cemented joint arthroplasty [5-9]. Despite similarities in pathophysiology, PBSS-related embolic events remain underreported, and data are lacking regarding their frequency, risk stratification, and outcomes, particularly in high-risk oncologic populations with cardiopulmonary comorbidities.

Here, we report a fatal case of cardiopulmonary collapse immediately following the balloon insufflation step of PBSS. This case is notable for its rapid and catastrophic course, despite being performed at skeletal sites commonly regarded as low risk (the humerus and ilium). We believe it highlights an underappreciated risk of embolic complications in the setting of intramedullary pressurization, particularly among elderly cancer patients with pulmonary hypertension or compromised cardiopulmonary reserve. To our knowledge, this is the first reported case involving extracorporeal membrane oxygenation (ECMO) initiation during PBSS. This report emphasizes the need for perioperative vigilance and potential protocol modifications to improve outcomes in this vulnerable population. The patient in the case report was deceased, and consent was obtained from her family.

## Case presentation

A 68-year-old female patient with a history of metastatic sarcoma underwent prophylactic stabilization of the right humerus using PBSS for sarcoma of the right humerus. She also had metastatic involvement of the lung and a remote history of deep vein thrombosis (DVT) and pulmonary embolism (PE). Her anticoagulant therapy with apixaban was held 48 hours prior to the operative procedure and was not bridged to enoxaparin or low-molecular-weight heparin. Her preoperative echocardiogram (ECHO) revealed normal left ventricular (LV) function and ejection fraction, mildly reduced right ventricular (RV) function, and an estimated RV systolic pressure of 66.1 mmHg. Preoperative laboratory evaluation revealed a hemoglobin level of 9.0 g/dL and platelet count of 112 × 10⁹/L. Basic metabolic panel and coagulation profile were within normal limits.

The patient had been on 2 L/min of supplemental oxygen via nasal cannula for the past month but denied experiencing any shortness of breath when using it. She was able to perform daily activities with assistance, with an estimated functional capacity of less than four metabolic equivalents of task (METs). On physical examination, she appeared in no acute distress and was alert and oriented. Respiratory effort was nonlabored while using a nasal cannula. Examination of the right upper extremity revealed a large mass on the posterior aspect of the arm. 

**Figure 1:**
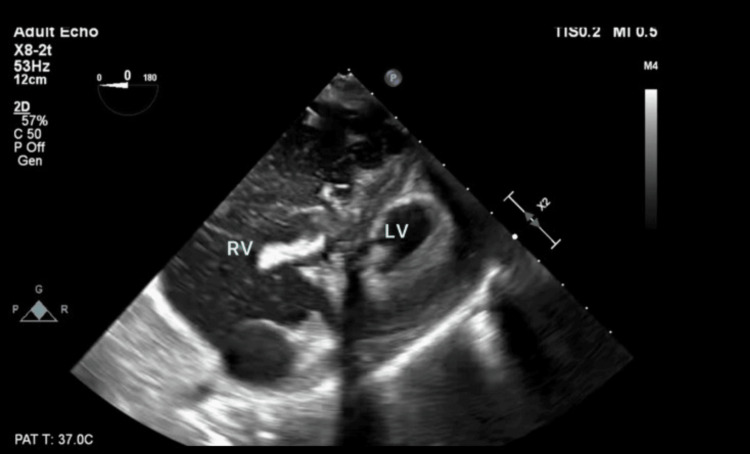
Trans-gastric basal short-axis view reveals a dilated right ventricle and a severely underfilled and hyperdynamic left ventricle concerning for pulmonary embolism. LV: Left ventricle; RV: right ventricle

Following an uneventful induction and intubation, a left radial arterial catheter was established. The procedure progressed without complications for approximately two hours. However, following the bone reaming and subsequent balloon inflation steps of the PBSS procedure, the patient experienced a sudden cardiopulmonary collapse characterized by pulseless electrical activity (PEA), hypoxia (SpO_2_ 40%), and hypotension (systolic blood pressure 30 mmHg). Cardiopulmonary resuscitation was initiated, achieving return of spontaneous circulation (ROSC) after one minute of advanced cardiac life support (ACLS) and one round of cardiopulmonary resuscitation (CPR). A transesophageal echocardiography (TEE) performed during resuscitation revealed a severely dilated RA and RV with an underfilled, hyperdynamic LV, suggestive of a massive PE (Figure 1, Video 1). Thrombolytic therapy was considered; however, it was not administered, as the clinical context and ongoing procedure suggested a higher likelihood of debris or fat embolism rather than DVT. Despite resuscitation efforts, the patient's condition continued to deteriorate, necessitating the initiation of emergent veno-arterial ECMO. The surgery was completed, and closure of the soft tissue and skin was performed following stabilization of the patient on ECMO. Postoperatively, the patient deteriorated and transitioned to comfort care on postoperative day five.

**Video 1:** Video clip of trans-gastric basal short-axis view reveals a dilated right ventricle and a severely underfilled and hyperdynamic left ventricle concerning for pulmonary embolism.

## Discussion

PBSS procedure

PBSS is a minimally invasive orthopedic surgical procedure. It involves canal preparation with a reamer over a guidewire, followed by bone canal reaming. A balloon catheter is then inserted into the medullary canal and infused with a photosensitive monomer under pressure, expanding to fill the canal and press against the cortices. The pressure required to insufflate the balloon is listed as “moderate” by the manufacturer; an exact pressure is not specified. Finally, a fiber-optic blue light induces polymerization and solidification of the monomer [1,5].

Fat embolism syndrome (FES) and bone cement implantation syndrome (BCIS), along with subsequent PE, are recognized complications of traditional arthroplasty [6-10]. However, the incidence of PE following humerus repair after trauma was 5.1% post-surgery [11], with even fewer cases reported involving the iliac bone repair. The dislodgement of the PBSS monomer or a fat embolus from the medullary canal during reaming the marrow has been reported following the PBSS procedure [3]. Sabharwal et al. described two cases of reported hemodynamic collapse during humerus stabilization with a photodynamic implant [3]. In this case, severe hemodynamic instability occurred shortly after balloon dilation and liquid monomer infusion. The patient also developed acute hypoxic respiratory failure and required four days of ventilatory support and several days of elevated oxygen requirements postoperatively. The authors speculated that in this instance, the complications were likely caused by the balloon insufflation-induced dislodgement of marrow and subsequent embolization.

PBSS is often chosen for older patients with metastatic metaphyseal involvement. Increasing age heightens the risk of osteoporosis and leads to marrow space being increasingly filled with fat due to a decrease in hematopoietic tissue [12]. Tumor invasion increases vascularity and can alter bone structure. Older patients also are at increased risk of pulmonary hypertension (PH) due to age-related factors and comorbidities like left heart disease, chronic lung disease, and thromboembolic disease. Aging causes structural changes in the pulmonary vasculature, leading to elevated pulmonary pressures [13]. In our case, the patient was elderly, had a history of metastatic cancer with significant pulmonary involvement, and exhibited signs of pulmonary hypertension and right heart strain. This reduced physiological reserve makes this patient more susceptible to adverse effects of an intraoperative pulmonary embolism, reducing a patient’s ability to tolerate an embolic load, leading to RV dysfunction [3,14,15].

We propose that bone reaming generates debris, including marrow, bone fragments, and blood clots due to localized bleeding. After balloon inflation, this debris is directed toward endosteal vessels and enters systemic circulation, potentially causing pulmonary embolism and cardiovascular collapse [3]. In addition, PBSS was indicated for an impending, pathologic fracture. In completed fractures, increased medullary pressure during monomer infusion might be relieved through the fracture site, acting as a "pop-off" valve. For impending fractures, the intact medullary canal may function as a closed system, leading to greater pressurization during balloon inflation [3]. Despite creating a distal vent hole intraoperatively to mitigate intramedullary pressure during balloon inflation, medullary contents (e.g., debris) can be pushed into systemic circulation, leading to massive pulmonary embolism and cardiovascular collapse.

In our case, debris embolism is the most likely cause of pulmonary embolism. Following reaming and canal clearing, residual marrow and bone fragments, as well as ongoing bleeding and reaccumulation of clots, may occur. The patient was hemodynamically stable during the procedure but experienced cardiopulmonary collapse following balloon insufflation. Unlike traditional orthopedic repairs, the PBSS procedure requires intramedullary pressurization. This may explain why locations such as the humerus and iliac bone, typically not associated with massive fat embolism can result in one [16]. The intact balloon in this case suggests debris embolism, rather than polymer embolization as the likely cause. DVT was considered, given the elevated risk in cancer patients and the fact that the patient was on apixaban. However, postoperative ultrasound and computed tomography did not show evidence of active DVT or PE.

Management recommendations

PBSS, though innovative and beneficial, can give rise to complications like debris, fat embolism, or polymer embolization. Though PE is typically associated with femur repair, it may also occur in the humerus and iliac bone repair. Patients with risk factors like pulmonary hypertension, RV dysfunction, LV failure, diastolic dysfunction, aortic or mitral valve disease, left atrial enlargement, PFO with right-to-left shunting, chronic obstructive pulmonary disease (COPD), and interstitial lung disease are at increased risk of not tolerating an intraoperative PE (Table 1). Additionally, patients over 65 years old face higher risks due to changes in the pulmonary vasculature [13].

**Table 1:** Identifying high-risk patients with reduced tolerance to intraoperative embolic events during the PBSS procedure [17]. LV: Left ventricle; RV, right ventricle; COPD: chronic obstructive pulmonary disease; PFO: patent foramen ovale.

High-Risk Comorbidities for Patients Undergoing PBSS
Significant Cardiac Disease
Pulmonary hypertension
RV failure
LV failure
Diastolic dysfunction
Aortic or mitral valve disease
PFO with evidence of right-to-left shunting
Significant Pulmonary Disease
COPD
Interstitial lung disease
Bronchiectasis
Primary or metastatic lung cancer
Increasing Age
Age >65 years

Following our experiences at our institution, we risk-stratify patients coming for the PBSS procedure. Perioperative management of high-risk cases involves invasive monitoring, a preoperative echo and bubble study ruling out PFO, and intraoperative TEE placement.

Prior to the procedure, ultrasound imaging is utilized to identify major venous outflow near the surgical site. If an embolus is suspected, compressing the vein can reduce and slow the embolic load to the heart and lungs, allowing for time for supportive care. For example, the femoral vein can be identified in femur reaming procedures and the axillary vein in upper extremity reaming cases, where a tourniquet can be used. A preoperative inferior vena cava (IVC) filter may be considered in high-risk cases, though its effectiveness at capturing small bone marrow emboli is limited.

Effective intraoperative team communication is crucial. Presurgical discussions may include evacuating the canal of marrow and tumor using suction before device insertion to reduce embolic load, creating a distal vent hole to lower intramedullary pressurization, and slowly infusing the monomer to prevent a sudden efflux of medullary contents [11]. Surgeons should communicate directly with the anesthesia team before monomer infusion and maintain ongoing communication throughout balloon inflation to ensure hemodynamic stability. 

In high-risk cases, a cardiothoracic anesthesiologist with TEE expertise, along with a cardiothoracic surgeon and perfusionist, may be needed for ECMO [18,19].

## Conclusions

PBSS offers a minimally invasive option for managing metastatic bone disease, but it poses real risks for patients with limited cardiopulmonary reserve. Even in anatomical sites typically seen as low-risk, such as the humerus or ilium, embolic events during canal reaming and balloon insufflation can lead to cardiopulmonary collapse. Identifying high-risk patients, particularly those with cardiac or pulmonary comorbidities, risk of systemic embolization, or advanced age, is essential. For these individuals, careful preoperative assessment, intraoperative planning, and close coordination between surgical and anesthesia teams are key. Practical steps include early imaging and monitoring, TEE use, arterial access, and preparation for rapid intervention with supportive therapies. This case underscores the importance of proactive, team-based strategies to mitigate the risk of serious intraoperative complications and improve outcomes in vulnerable patients undergoing PBSS.

## References

[1] (2024). IlluminOss Medical. Surgical Technical Guide: Humerus, radius and ulna. https://illuminoss.com/uploads/nuggets/5eaa859f7ee3385fc186902b/2019-All-forearm-surgical-technique-.pdf.

[2] Zoccali C, Attala D, Pugliese M, di Uccio AS, Baldi J (2021). The IlluminOss® photodynamic bone stabilization system for pathological osteolyses and fractures of the humerus: indications, advantages and limits in a series of 12 patients at 24 months of minimum follow-up. BMC Musculoskelet Disord.

[3] Sabharwal S, Boland PJ, Vaynrub M (2024). Severe hemodynamic collapse during humerus stabilization with photodynamic implant: a report of two cases. JBJS Case Connect.

[4] Zyskowski M, Wurm M, Greve F (2022). A prospective randomized study comparing functional outcome in distal fibula fractures between conventional AO semitubular plating and minimal invasive intramedullary "photodynamic bone stabilisation". J Clin Med.

[5] Gausepohl T, Pennig D, Heck S, Gick S, Vegt PA, Block JE (2017). Effective management of bone fractures with the Illuminoss® Photodynamic Bone Stabilization System: initial clinical experience from the European Union Registry. Orthop Rev (Pavia).

[6] Arcelus JI, Kudrna JC, Caprini JA (2006). Venous thromboembolism following major orthopedic surgery: what is the risk after discharge?. Orthopedics.

[7] (2024). Bone cement implantation syndrome. Anaesthesia Tutorial of the Week (ATOTW). https://resources.wfsahq.org/atotw/bone-cement-implantation-syndrome.

[8] Lempert M, Halvachizadeh S, Ellanti P, Pfeifer R, Hax J, Jensen KO, Pape HC (2021). Incidence of fat embolism syndrome in femur fractures and its associated risk factors over time - a systematic review. J Clin Med.

[9] Pitto RP, Koessler M, Kuehle JW (1999). Comparison of fixation of the femoral component without cement and fixation with use of a bone-vacuum cementing technique for the prevention of fat embolism during total hip arthroplasty. A prospective, randomized clinical trial. J Bone Joint Surg Am.

[10] Koval KJ, Zuckerman JD (2009). Perioperative considerations in geriatric patients with hip fracture. J Orthop Trauma.

[11] Hoxie SC, Sperling JW, Cofield RH (2007). Pulmonary embolism after operative treatment of proximal humeral fractures. J Shoulder Elbow Surg.

[12] Longo DL (2008). Bone marrow in aging: changes? Yes; clinical malfunction? Not so clear. Blood.

[13] Dharma S, Kedev S, Jukema JW (2013). Thrombus management in the catheterisation laboratory in the setting of primary percutaneous coronary intervention: what is the current evidence?. Heart.

[14] Kindler CH, Evgenov OV, Crawford LC (2019). Anesthesia for orthopedic surgery. Miller’s Anesthesia.

[15] Hope WW, Demeter BL, Newcomb WL, Schmelzer TM, Schiffern LM, Heniford BT, Sing RF (2007). Postoperative pulmonary embolism: timing, diagnosis, treatment, and outcomes. Am J Surg.

[16] Davies MG, Hart JP (2023). Current status of ECMO for massive pulmonary embolism. Front Cardiovasc Med.

[17] Klein AA, Skubas NJ, Ender J (2014). Controversies and complications in the perioperative management of patients undergoing orthopedic surgery. Curr Opin Anaesthesiol.

[18] Maitre S (2006). Causes, clinical manifestations, and treatment of fat embolism. Virtual Mentor.

[19] Deng T, Xu K, Wu B, Sheng F, Li X, Zhu Z, Zhang Z (2022). Clinical characteristics and risk factors predictive of pulmonary embolism complicated in bronchiectasis patients: a retrospective study. BMC Pulm Med.

